# Association between duration of smoking abstinence before non-small-cell lung cancer diagnosis and survival: a retrospective, pooled analysis of cohort studies

**DOI:** 10.1016/S2468-2667(23)00131-7

**Published:** 2023-09

**Authors:** Aline F Fares, Yao Li, Mei Jiang, M Catherine Brown, Andrew C L Lam, Reenika Aggarwal, Sabine Schmid, Natasha B Leighl, Frances A Shepherd, Zhichao Wang, Nancy Diao, Angela S Wenzlaff, Juntao Xie, Takashi Kohno, Neil E Caporaso, Curtis Harris, Hongxia Ma, Matthew J Barnett, Leticia Ferro Leal, G Fernandez-Tardon, Mónica Pérez-Ríos, Michael P A Davies, Fiona Taylor, Ben Schöttker, Paul Brennan, David Zaridze, Ivana Holcatova, Jolanta Lissowska, Beata Świątkowska, Dana Mates, Milan Savic, Hermann Brenner, Angeline Andrew, Angela Cox, John K Field, Alberto Ruano-Ravina, Sanjay S Shete, Adonina Tardon, Ying Wang, Loic Le Marchand, Rui Manuel Reis, Matthew B Schabath, Chu Chen, Hongbing Shen, Brid M Ryan, Maria Teresa Landi, Kouya Shiraishi, Jie Zhang, Ann G Schwartz, Ming S Tsao, David C Christiani, Ping Yang, Rayjean J Hung, Wei Xu, Geoffrey Liu

**Affiliations:** Princess Margaret Cancer Centre and University Health Network (A F Fares MD, M Jiang PhD, M Catherine Brown MSc, A C L Lam MD, R Aggarwal MPH, S Schmid MD, Prof N B Leighl MD, Prof F A Shepherd MD, Prof M S Tsao PhD, Prof W Xu PhD, Prof G Liu MD) and Dalla Lana School of Public Health (Y Li MSc, Prof R J Hung PhD, Prof W Xu, Prof G Liu), University of Toronto, Toronto, ON, Canada; Division of Medical Oncology, Faculty of Medicine of São José do Rio Preto, São Paulo, Brazil (A F Fares); State Key Laboratory of Respiratory Disease, National Clinical Research Center for Respiratory Disease, Guangzhou Institute of Respiratory Health, First Affiliated Hospital of Guangzhou Medical University, Guangzhou, China (M Jiang); Universitätsklinik für Medizinische Onkologie, Inselspital Bern, Bern, Switzerland (S Schmid); Department of Health Sciences Research, Mayo Clinic, Rochester, MN, USA (Z Wang MD, Prof P Yang MD); Division of Pulmonary and Critical Care Medicine, Jiangsu Province Hospital of Chinese Medicine, Affiliated Hospital of Nanjing University of Chinese Medicine, Nanjing, China (Z Wang); Harvard T H Chan School of Public Health, Harvard University, Boston, MA, USA (N Diao ScD, Prof D C Christiani MD); Barbara Ann Karmanos Cancer Institute, Wayne State University, Detroit, MI, USA (A S Wenzlaff MPH, Prof A G Schwartz PhD); Department of Thoracic Surgery, Fudan University Shanghai Cancer Center, Shanghai, China (J Xie MD, Prof J Zhang MD); Division of Genome Biology (Prof T Kohno PhD, Prof K Shiraishi PhD), Department of Clinical Genomics (Prof K Shiraishi), National Cancer Center Research Institute, Tokyo, Japan; National Cancer Institute (Prof N E Caporaso MD, Prof M T Landi MD) and Laboratory of Human Carcinogenesis, Centre for Cancer Research (Prof C Harris MD, B M Ryan PhD), National Institutes of Health, Bethesda, MD, USA; Department of Epidemiology, School of Public Health, Nanjing Medical University, Nanjing, China (H Ma PhD, Prof H Shen PhD); Public Health Sciences, Biostatistics Program, Fred Hutchinson Cancer Center, Seattle, WA, USA (M J Barnett MSc); Molecular Oncology Research Center, Barretos Cancer Hospital, Barretos, Brazil (L F Leal PhD, Prof R M Reis PhD); University Institute of Oncology of Asturias–Cajastur Social Programme, University of Oviedo, Oviedo, Spain (G Fernandez-Tardon PhD, Prof A Tardon PhD); Health Research Institute of Asturias, Oviedo, Spain (G Fernandez-Tardon, Prof A Tardon); Consorcio de Investigación Biomédica en Red de Epidemiología y Salud Pública, Oviedo, Spain (G Fernandez-Tardon, Prof A Tardon); Department of Preventive Medicine and Public Health, University of Santiago de Compostela, Santiago de Compostela, Spain (M Pérez-Ríos PhD, Prof A Ruano-Ravina); Roy Castle Lung Cancer Research Programme, Department of Molecular and Clinical Cancer Medicine, University of Liverpool, Liverpool, UK (M P A Davies PhD, Prof J K Field PhD); Department of Oncology and Metabolism, University of Sheffield, Sheffield, UK (F Taylor MBChB, Prof A Cox PhD); Weston Park Cancer Centre, Sheffield Teaching Hospital Foundation Trust, Sheffield, UK (F Taylor); Division of Clinical Epidemiology and Aging Research (B Schöttker PhD, Prof H Brenner MD), Division of Preventive Oncology (Prof H Brenner), National Center for Tumor Diseases (Prof H Brenner), and German Cancer Consortium (Prof H Brenner), German Cancer Research Center, Heidelberg, Germany; Network of Aging Research, Heidelberg University, Heidelberg, Germany (B Schöttker); Genomic Epidemiology Branch, International Agency for Research on Cancer, Lyon, France (Prof P Brennan PhD); N N Blokhin National Medical Research Centre of Oncology, Moscow, Russia (Prof D Zaridze DSc); Institute of Public Health and Preventive Medicine and Department of Oncology, Second Faculty of Medicine and University Hospital Motol, Charles University, Prague, Czech Republic (Prof I Holcatova MD); Department of Cancer Epidemiology and Prevention, M Sklodowska-Curie National Research Institute of Oncology, Warsaw, Poland (Prof J Lissowska PhD); Nofer Institute of Occupational Medicine, Łódź, Poland (Prof B Świątkowska PhD); National Institute of Public Health, Bucharest, Romania (Prof D Mates MD); Department of Thoracic Surgery, Clinical Center of Serbia, Belgrade, Serbia (Prof M Savic MD); Dartmouth-Hitchcock Medical Center, Lebanon, NH, USA (Prof A Andrew PhD); Health Research Institute of Santiago de Compostela, Santiago de Compostela, Spain (Prof A Ruano-Ravina PhD); M D Anderson Cancer Center, University of Texas, Houston, TX, USA (Prof S S Shete PhD); American Cancer Society, Atlanta, GA, USA (Prof Y Wang PhD); University of Hawai’i Cancer Centre, University of Hawai’i, Honolulu, HI, USA (Prof L Le Marchand MD); Life and Health Sciences Research Institute, Medical School, University of Minho, Braga, Portugal (Prof R M Reis); Life and Health Sciences Research Institute–Biomaterials, Biodegradables and Biomimetics Research Group Associate Laboratory, Braga, Portugal (Prof R M Reis); H Lee Moffitt Cancer Center and Research Institute, Tampa, FL, USA (Prof M B Schabath PhD); Program in Epidemiology, Cancer Prevention Program, Fred Hutchinson Cancer Center, Seattle, WA, USA (Prof C Chen PhD); LunenfeldTanenbaum Research Institute, Sinai Health Systems, Toronto, ON, Canada (Prof R J Hung)

## Abstract

**Background:**

The association between duration of smoking abstinence before non-small-cell lung cancer (NSCLC) diagnosis and subsequent survival can influence public health messaging delivered in lung-cancer screening. We aimed to assess whether the duration of smoking abstinence before diagnosis of NSCLC is associated with improved survival.

**Methods:**

In this retrospective, pooled analysis of cohort studies, we used 26 cohorts participating in Clinical Outcomes Studies of the International Lung Cancer Consortium (COS-ILCCO) at 23 hospitals. 16 (62%) were from North America, six (23%) were from Europe, three (12%) were from Asia, and one (4%) was from South America. Patients enrolled were diagnosed between June 1, 1983, and Dec 31, 2019. Eligible patients had smoking data before NSCLC diagnosis, epidemiological data at diagnosis (obtained largely from patient questionnaires), and clinical information (retrieved from medical records). Kaplan-Meier curves and multivariable Cox models (ie, adjusted hazard ratios [aHRs]) were generated with individual, harmonised patient data from the consortium database. We estimated overall survival for all causes, measured in years from diagnosis date until the date of the last follow-up or death due to any cause and NSCLC-specific survival.

**Findings:**

Of 42 087 patients with NSCLC in the COS-ILCCO database, 21 893 (52·0%) of whom were male and 20 194 (48·0%) of whom were female, we excluded 4474 (10·6%) with missing data. Compared with current smokers (15 036 [40·0%] of 37 613), patients with 1–3 years of smoking abstinence before NSCLC diagnosis (2890 [7·7%]) had an overall survival aHR of 0·92 (95% CI 0·87–0·97), patients with 3–5 years of smoking abstinence (1114 [3·0%]) had an overall survival aHR of 0·90 (0·83–0·97), and patients with more than 5 years of smoking abstinence (10 841 [28·8%]) had an overall survival aHR of 0·90 (0·87–0·93). Improved NSCLC-specific survival was observed in 4301 (44%) of 9727 patients who had quit cigarette smoking and was significant at abstinence durations of more than 5 years (aHR 0·87, 95% CI 0·81–0·93). Results were consistent across age, sex, histology, and disease-stage distributions.

**Interpretation:**

In this large, pooled analysis of cohort studies across Asia, Europe, North America, and South America, overall survival was improved in patients with NSCLC whose duration of smoking abstinence before diagnosis was as short as 1 year. These findings suggest that quitting smoking can improve overall survival, even if NSCLC is diagnosed at a later lung-cancer screening visit. These findings also support the implementation of public health smoking cessation strategies at any time.

## Introduction

With 1·3 billion people who currently smoke worldwide, cigarette smoking is the greatest, single, modifiable risk factor for all-cause mortality.^1,2^ With more than 70 carcinogens, cigarette smoking is associated with the development of many cancers, but is most closely associated with lung cancer,^3^ for which the population-attributable risk is large.^4^

Lung cancer is the leading cause of cancer death worldwide.^5^ Non-small-cell lung cancer (NSCLC) comprises more than 80% of all lung cancers,^6^ with an overall 5-year survival rate of 28% in the USA.^7^ Smoking also affects lung-cancer prognosis;^8^ survival outcomes are best for people who have never smoked, worst for current smokers, and are between these two groups for former smokers.^9,10^ In the general population, lung-cancer risk is reduced by half after 5–10 years of smoking abstinence.^8,11,12^ Smoking abstinence also reduces the risk of cardiovascular and respiratory disease events, the risks of several other cancer types (eg, cancers of the oral cavity and oesophagus), and improves reproductive health.^13^

Lung-cancer screening with low-dose computed tomography (LDCT)^14,15^ offers health professionals opportunities to include smoking-cessation components.^16–19^ After lung-cancer screening, participants have improved readiness and motivation to quit smoking.^20^ All benefits of smoking cessation should be explained to people who smoke during this moment to improve the chances of successful smoking abstinence.^21^

Contrary to the well known effects on cancer prevention by smoking cessation,^11^ the prognostic association between improved outcomes and smoking cessation for people who eventually develop lung cancer remains under-recognised.^8,12,22^ Continuing to smoke after lung-cancer diagnosis negatively affects treatment effectiveness by increasing the clearance of systemic therapies and restricting the effectiveness of radiation.^23^ However, some current smokers are not aware of the benefits of smoking cessation.^24^

In this analysis of Clinical Outcomes Studies of the International Lung Cancer Consortium (COS-ILCCO), we aimed to assess whether the duration of smoking abstinence before NSCLC diagnosis is associated with improved overall survival—and if so, the shortest effective duration of this abstinence—to provide evidence supporting the multifaceted benefits of smoking cessation that might increase the motivation of people to stop smoking and strengthen the delivery from providers of smoking-cessation messaging. We also aimed to assess the association between smoking abstinence and NSCLC-specific survival, as motivation to quit smoking might be improved if there is a direct link between smoking abstinence and lung-cancer survival specifically.

## Methods

### Study design and participants

For this retrospective, pooled analysis of cohort studies, individual-level data were pooled from patients with NSCLC across 26 studies participating in COS-ILCCO at 23 hospitals from the USA (n=15), China (n=2), the UK (n=2), Spain (n=2), Brazil (n=1), Canada (n=1), Germany (n=1), Japan (n=1), Russia (n=1), Czech Republic (n=1), Poland (n=1), Romania (n=1), and Serbia (n=1; appendix p 3). These studies included patients diagnosed between June 1, 1983, and Dec 31, 2019. Each study was approved by local institutional research ethics boards.

Methods of recruitment, selection, and follow-up are reported in the cohort studies (appendix p 3). Eligible patients had smoking data before lung-cancer diagnosis, epi demiological data at diagnosis (ie, age, sex, ethnicity, and education), and clinical information (ie, histology, date of diagnosis, disease stage at diagnosis, vital status at last follow-up, and date of either last follow-up or death). Data across studies were checked for inconsistency, inadmissible values, and outliers before harmonising and uniformly coding variables into a common dataset.^25,26^ Written informed consent was obtained from all study participants by the coordinators of the 26 original cohorts.

### Variables

Covariates considered in this analysis (ie, age at diagnosis, sex, ethnicity, cumulative smoking measured in pack years, education, disease stage at diagnosis, histology, and year of diagnosis) were available in the consortium database and were identified as being prognostically important. The year of diagnosis was a surrogate measure of global overall improvement in clinical management and overall survival over time.

### Data collection

Self-reported smoking behaviour at lung-cancer diagnosis was collected across all studies, if possible. The final analysis included all studies, but we also conducted a sensitivity analysis excluding studies that did not collect either pack years or smoking information at time of diagnosis (baseline). Smoking status was coded as people who had never smoked (ie, who had smoked fewer than 100 cigarettes in their lifetime), current smokers (ie, had smoked cigarettes within 1 year of NSCLC diagnosis date), and former smokers (ie, had quit at least 1 year before diagnosis). 18 consortium studies were originally epidemiological case-control studies in which 1 year before the date of diagnosis was a cutoff to define current versus former smokers; we kept the same definition for our prognostic analyses. For former smokers, we included only people for whom the date of their last cigarette was reported or a time interval from the date of quitting smoking until the date of NSCLC diagnosis. These data were used to calculate smoking-abstinence duration. Date of diagnosis, vital status, overall survival, cause of death, histology, and stage were based on medical records; age, ethnicity, sex (offered options were male or female), and education level were self-reported.

### Statistical analysis

Descriptive statistics included frequencies and percentages for categorical variables and medians and IQRs for continuous variables. χ² tests were used to compare categorical variables and Kruskal-Wallis tests were used to compare continuous variables.

The primary objective was overall survival for all causes, measured in years from diagnosis date until the date of the last follow-up or death due to any cause, in all participating patients with NSCLC. We generated unadjusted and adjusted overall-survival Kaplan-Meier curves and log-rank tests to support previously shown associations between smoking status and survival,^27^ and to evaluate survival effects by smoking status or length of smoking abstinence. Cox proportional hazards regression models^28^ were adjusted for clinicoepidemiological factors that were identified in a baseline clinical multivariable model. Among people who had ever smoked, we used current smokers as the reference group for analyses evaluating unadjusted hazard ratios (HRs) and adjusted hazard ratios (aHRs) of former smokers for associations with duration of smoking abstinence before NSCLC diagnosis (ie, the primary analysis).

Because the primary analysis pooled all patients with a length of smoking abstinence of more than 5 years before NSCLC diagnosis, we also sought to explore the association between various durations of long-term smoking abstinence and NSCLC survival. We generated adjusted smoothed penalised spline curves of people who had ever smoked to visually compare the overall-survival aHR of long-term smoking abstinence (plotted as a continuous variable) before NSCLC diagnosis compared with current smokers. Smoothed penalised spline curves described the aHR associations between smoking status, long-term smoking abstinence, and NSCLC-diagnosis date. Exploratory analyses also evaluated subgroups of patients via clinicodemographic variables, reported in Forest plots. Furthermore, we assessed NSCLC-specific survival as a secondary outcome using a proportional hazards model for the sub-distribution of a competing risk of death.^29^ For both continuous and categorical variables, participants with missing data were excluded from the analyses. Participants with missing data on education were classified under the other education category. When loss to follow-up occurred, survival analysis with right-censoring was used.

To provide examples of absolute risk benefit, we generated 5-year and 10-year overall-survival estimates by smoking status and duration of smoking abstinence for a prototypical White, male patient with stage 1 lung adenocarcinoma who was younger than 65 years with less than 40 pack years of smoking history and being treated at the Mayo Clinic (Rochester, MI, USA), which was the study site with the largest patient cohort. We also generated cumulative-incidence estimates of NSCLC-specific survival at 5 years and at 10 years for the same prototypical male patient treated at the Princess Margaret Cancer Center (Toronto, ON, Canada). The Mayo Clinic cohort was excluded from this secondary analysis owing to a high proportion of missing data for NSCLC-specific survival.

Sensitivity analyses were done to assess the robustness of our results (appendix pp 10–13). All analyses were conducted with R version 4.2.2 and SAS version 9.4. All p values were based on two-sided tests; the primary and secondary analyses were considered statistically significant at p<0·05.

### Role of the funding source

The funders of this analysis had no role in study design, data collection, data analysis, data interpretation, writing of the report, or the decision to submit for publication.

## Results

Of the 26 studies participating in COS-ILCCO, 16 (62%) were from North America, six (23%) were from Europe, three (12%) were from Asia, and one (4%) was from South America (appendix p 3). Of 42 087 patients with NSCLC in the COS-ILCCO database, 21 893 (52·0%) of whom were male and 20 194 (48·0%) of whom were female, we excluded 4474 (10·6%) with missing data (588 [13·1%] with missing information on smoking status and 3886 [86·9%] with missing information on length of smoking abstinence). As a result, we analysed 37 613 patients with NSCLC; 7732 (20·6%) people who had never smoked, 15 036 (40·0%) current smokers, and 14 845 (39·5%) former smokers (2890 [7·7%] patients with 1–3 years of smoking abstinence, 1114 [3·0%] patients with 3–5 years of smoking abstinence, and 10 841 [28·8%] patients with >5 years of smoking abstinence; appendix p 2).

Clinicodemographic information of individuals who were analysed versus the full dataset and by smoking status of the analysed dataset are provided in the appendix (pp 4–5). Self-reported smoking behaviour at lung-cancer diagnosis was provided in 22 (85%) studies; two cohort studies (1819 [5%] of 37 613 patients) collected smoking information before the lung-cancer diagnosis date and two cohort studies (1029 [3%] of 37 613 patients) did not report pack-years information. Relative to current smokers or former smokers, people who had never smoked were more likely to be female, Asian, and diagnosed with adenocarcinomas (appendix p 5). As most studies conducted in Asia did not report education data (appendix p 6), missing education data were also associated with never smoking; this approach was taken consistently throughout the analysis. Compared with current smokers, former smokers were more likely to be older, have adenocarcinomas, have stage 1 (*vs* stage 4) NSCLC, and have lower cumulative smoking exposure (appendix p 5).

In the primary analysis of the association between duration of smoking abstinence and overall survival among people who had ever smoked, we first created clinical prognostic univariable and multivariable Cox proportional-hazard models that did not include duration of smoking abstinence, with or without smoking status, which were the basis for all subsequent multivariable analyses (appendix p 9). In these models, patients who were older, male, White, had less education, had a more advanced disease stage, had non-adenocarcinoma subtype lung cancers, and had greater cumulative smoking exposure were individually associated with significantly worse overall survival (appendixp 9). We then added the duration of smoking abstinence into these models, which replaced smoking status (table 1). Compared with current smokers, patients with 1–3 years of smoking abstinence before NSCLC diagnosis had an overall survival aHR of 0·92 (95% CI 0·87–0·97), patients with 3–5 years of smoking abstinence had an overall survival aHR of 0·90 (0·83–0·97), and patients with more than 5 years of smoking abstinence had an overall survival aHR of 0·90 (0·87–0·93).

**Figure 1:**
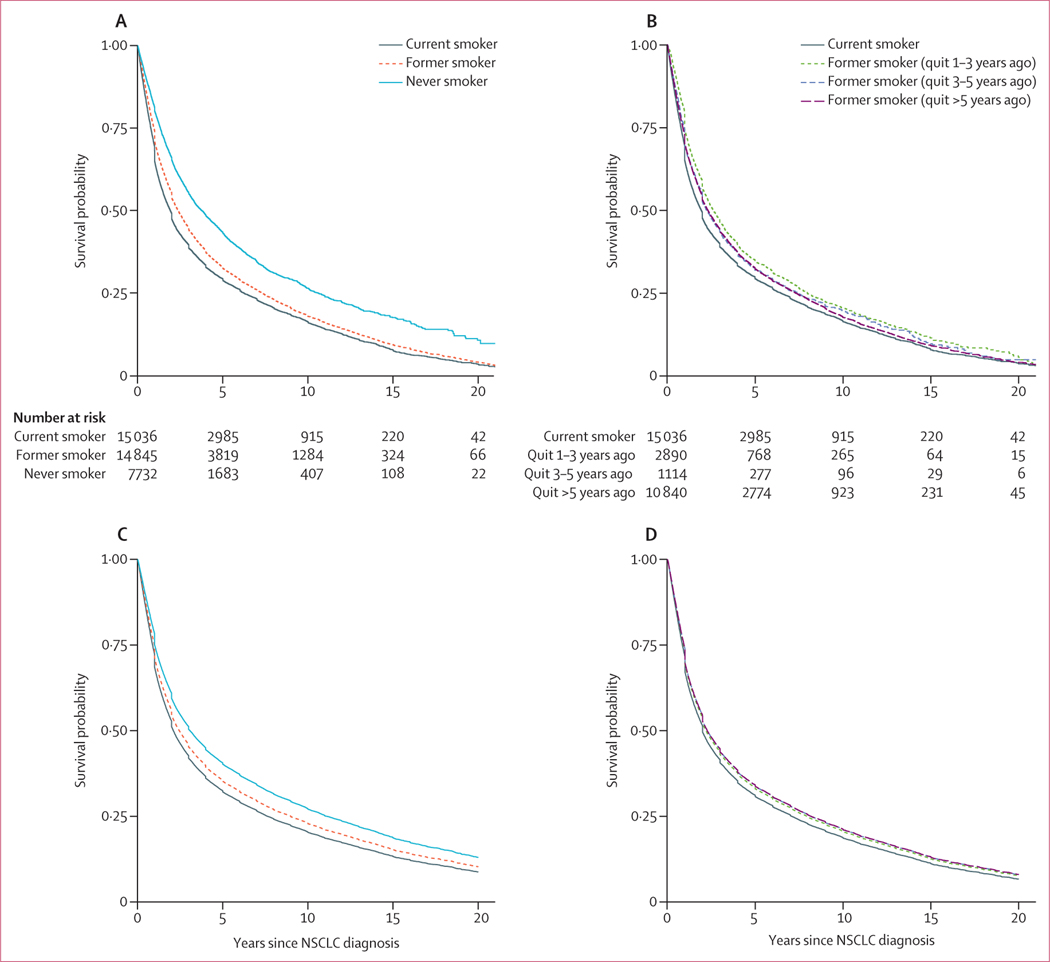
Overall survival curves among subgroups of patients with NSCLC by smoking status and by duration of smoking abstinence among people who have ever smoked (A) Unadjusted Kaplan-Meier curve by smoking status. (B) Unadjusted Kaplan-Meier curve among people who have ever smoked. (C) Fully adjusted survival curve by smoking status, estimated by Cox proportional-hazards model and adjusted by age, sex, ethnicity, educational status, clinical stage at diagnosis, study site, year of diagnosis, and histology. (D) Fully adjusted survival curve among people who have ever smoked, estimated by Cox proportional-hazards model and adjusted by age, sex, ethnicity, education, clinical stage at diagnosis, study site, year of diagnosis, histology, and pack years. Number at risk are provided for unadjusted survival curves. The p values of (A) and (B) are based on log-rank tests; the p-values of (C) and (D) are based on likelihood-ratio tests. NSCLC=non-small-cell lung cancer.

**Figure 2:**
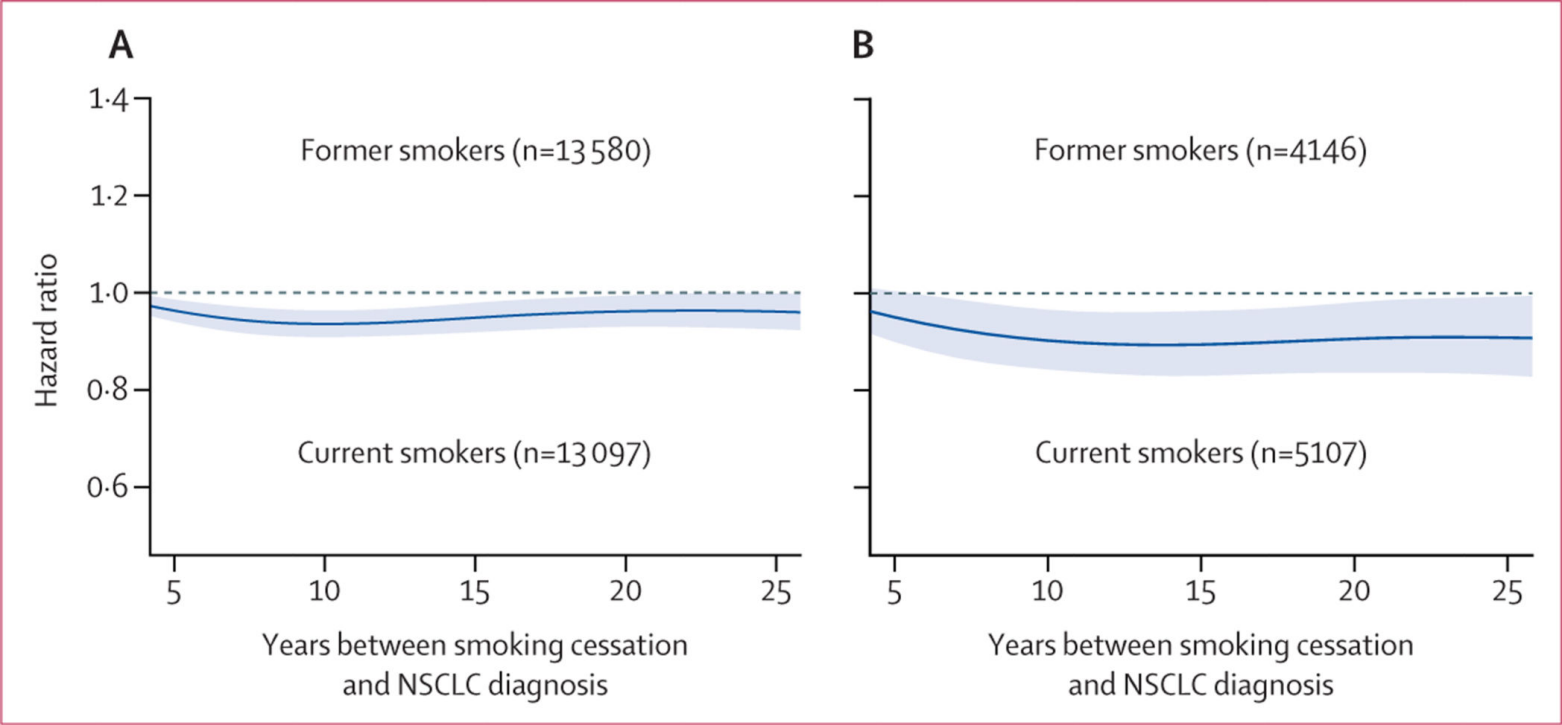
Association of long-term smoking abstinence (5–25 years) before a diagnosis of NSCLC with overall survival or NSCLC cancer-specific survival (A) Smoothed penalised spline curve showing hazard ratios of overall survival comparing former and current smokers. (B) Smoothed penalised spline curve showing hazard ratios of NSCLS-specific survival comparing former and current smokers. Former and current smokers are plotted against years of smoking abstinence. NSCLC=non-small-cell lung cancer.

Unadjusted and adjusted Kaplan-Meier curves (figure 1) substantiated previously shown relationships between smoking status and overall survival. People who had never smoked had longer survival than former smokers, who had longer survival than current smokers (p<0·0001; appendix p 7). From the smoothed penalised spline curves (figure 2), aHRs consistently showed a reduced risk at 5–25 years of smoking abstinence before NSCLC diagnosis.

Estimates for the probability of being alive and for the cumulative incidence of death specifically from NSCLC at 5 years and at 10 years for our prototypical White, male patient with stage 1 lung adenocarcinoma who was younger than 65 years, by smoking status, are provided in the appendix (p 8). For this prototypical patient, the probability of being alive 5 years after NSCLC diagnosis was 53% (95% CI 51–55) if he was a current smoker, 57% (55–59) if he was a former smoker, and 63% (61–65) if he had never smoked.

In this same prototypical patient, we compare being a current smoker with being a former smoker with various lengths of smoking abstinence (appendix p 8). The probability of being alive at 5 years was 54% (51–56) and at 10 years was 36% (33–39) if he was a current smoker. If he had more than 5 years of smoking abstinence before lung-cancer diagnosis, the probability of being alive at 5 years was 57% (55–60) and at 10 years was 40% (37–43).

The probability of being alive 10 years after NSCLC diagnosis was 35% (33–37) if he was a current smoker, 40% (37–42) if he was a former smoker, and 46% (44–49) if he had never smoked. In terms of NSCLC cumulative incidence of death at 5 years, this prototypical patient had estimates of 21% (13–28) if he was a current smoker, 19% (12–26) if he was a former smoker, and 17% (11–23) if he had never smoked. In terms of NSCLC cumulative incidence of death at 10 years, this patient had estimates of 26% (17–35) if he was a current smoker, 23% (15–31) if he was a former smoker, and 21% (13–28) if he had never smoked.

**Figure 3:**
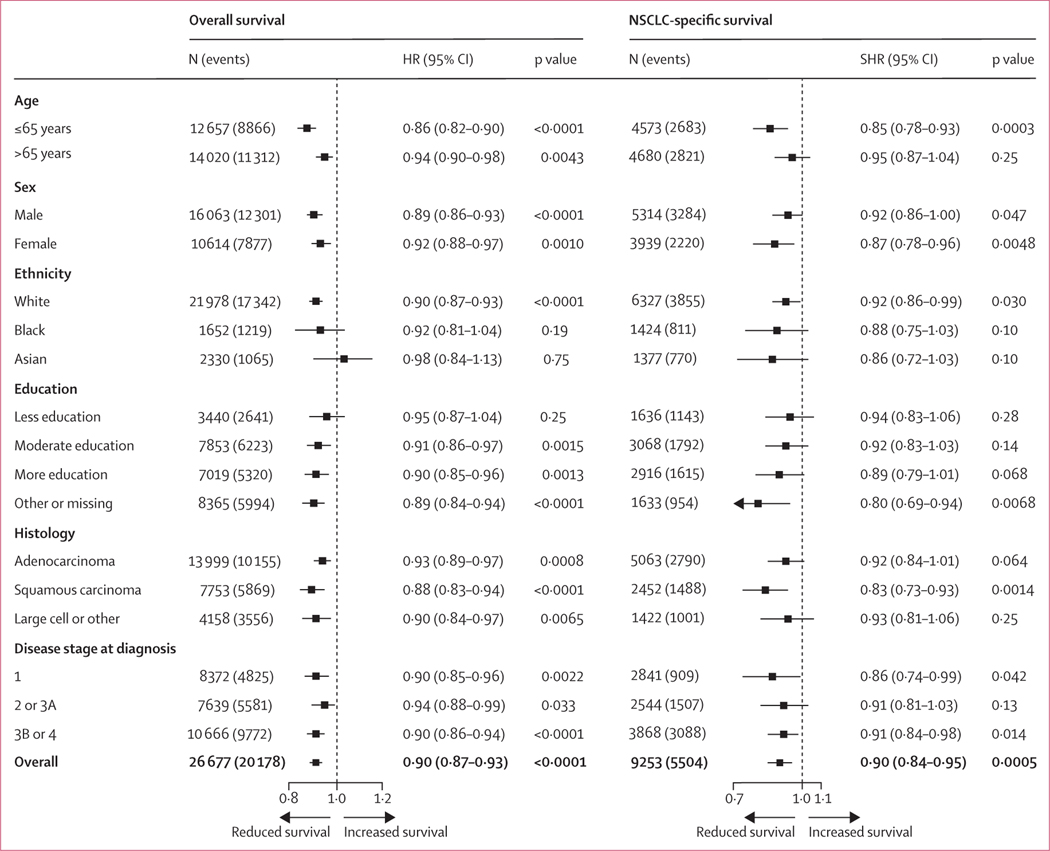
Forest plots of adjusted overall-survival HR and adjusted SHR for NSCLC-specific survival of former smokers with more than 1 year of smoking abstinence before NSCLC diagnosis versus current smokers with less than 1 year of smoking abstinence before NSCLC diagnosis HR=hazard ratio. NSCLC=non-small-cell lung cancer. SHR=sub-distribution hazard ratio.

In subgroup analyses of clinicodemographic factors (figure 3), although there was variability in the magnitude of association, most subgroups showed some improved overall survival after 1 year or more of smoking abstinence before NSCLC diagnosis compared with patients who were current smokers or who had less than 1 year of smoking abstinence before NSCLC diagnosis. Results of the sensitivity analyses excluding participants with no smoking information at diagnosis or pack years and current smokers who smoked fewer than two cigarettes every day showed similar effect sizes as the primary analysis (appendix pp 10–12).

9727 (32·6%) of 29 881 people who had ever smoked from 13 studies had available cause-of-death data. NSCLC cancer-specific survival was significantly improved for former smokers (*vs* current smokers) when the smoking-abstinence period was more than 5 years (aHR 0·87, 95% CI 0·81–0·93, p=0·0001). When the abstinence period was 1–3 years, the aHR was 0·94 (0·87–1·02, p=0·15) or when the smoking-abstinence period was 3–5 years it was 0·91, (0·79–1·05, p=0·21; table 2). NSCLC cancer-specific survival was improved in some clinicodemographic subgroups when smoking abstinence before NSCLC diagnosis was at least 1 year. Smoothed penalised spline curve analyses also showed a consistent improvement in cancer-specific survival when smoking abstinence before NSCLC diagnosis was between 5 and 25 years (figure 2B). Cumulative-incidence estimates of NSCLC-specific mortality at 5 years and at 10 years for our prototypical White, male patient with different smoking-abstinence durations are provided in the appendix (p 8). For example, if our prototypical patient was a current smoker at the time of NSCLC diagnosis, the cumulative incidence of death from NSCLC would be 27% (24–30) at 5 years and 34% (30–37) at 10 years. If this prototypical patient had a smoking-abstinence period of more than 5 years before diagnosis, the cumulative incidence of death from NSCLC would be 24% (21–27) at 5 years and 30% (26–33) at 10 years.

Subgroup analysis by cumulative smoking (ie, pack years) is shown in the appendix (pp 14–15). Regardless of how patients were coded with different pack-year cutoffs, there was a consistent directional pattern of improved overall survival across most smoking-abstinence durations in former smokers compared with current smokers. However, improved cancer-specific survival was seen only in former smokers with more than 5 years of smoking abstinence compared with current smokers.

We also conducted stratified analyses by decade of diagnosis. Larger benefits of smoking abstinence were seen in the 2000s (aHR 0·90, 95% CI 0·87–0·94, p<0·0001 for overall survival; 0·87, 0·81–0·93, p<0·0001 for NSCLC-specific survival) and 2010s (0·87, 0·80–0·94, p=0·0008 for overall survival; 0·79, 0·65–0·96, p=0·016 for NSCLC-specific survival) than in the 1980s (1·01, 0·41–2·47, p=0·98 for overall survival; 1·08, 0·93–1·27, p=0·31 for NSCLC-specific survival) and 1990s (0·94, 0·87–1·00, p=0·054 for overall survival; 1·08, 0·93–1·27, p=0·31 for NSCLC-specific survival; appendix p 16). We evaluated heterogeneity by study for both overall survival and cancer-specific survival (appendix pp 17–18). HRs from a meta-analysis with random-effects models were similar to the overall aHRs for overall survival and NSCLC-specific survival.

## Discussion

In this large, retrospective, pooled analysis of cohort studies, smoking abstinence for a duration as short as 1 year before NSCLC diagnosis was associated with improved overall survival compared with individuals who continued to smoke until NSCLC diagnosis. These findings were consistent across all age distributions, both sexes, multiple lung-cancer stages at diagnosis, and main histological subtypes of NSCLC, thereby supporting the statement that quitting smoking now can improve outcomes later, if later is defined as being at least 1 year.

Other, smaller studies^30^ that assessed various durations of smoking abstinence in former smokers versus current smokers found little evidence or non-significant aHRs for overall survival with effect sizes consistent with our results. Other, previous studies could be included in this analyses due to their participation in ILCCO.^31,32^ The strength of our analysis is the large number of patients with NSCLC across multiple continents, with exploratory analyses that showed that the directions of these associations were consistent across multiple demo graphic patient subgroups and clinical conditions at diagnosis. Also, compared with previous analyses of screening populations,^14,15^ our COS-ILCCO dataset contained a wider distribution of smokers by cumulative smoking exposure, with patients with NSCLC included from multiple continents and no restrictions on age or being asymptomatic at the time of lung-cancer diagnosis, which are eligibility criteria that are typically used for entry into screening programmes. Therefore, our findings are generalisable to a broader range of patients than these previous analyses, which is useful for smoking-cessation counselling outside of the low-dose CT screening context.

Although seemingly small, the observed effect size in overall survival and cancer-specific survival as a result of smoking abstinence before lung-cancer diagnosis is similar to receiving 3 months or 4 months of adjuvant chemotherapy in early-stage, resected NSCLC, which has an aHR of 0·89.^33^ This suggests the relative importance of our findings and the importance of encouraging smoking abstinence alongside adjuvant treatments.

Our results also provide evidence that the association between smoking abstinence and improved overall survival is not only due to reduced mortality from non-lung cancer causes, but also that with increasing smoking abstinence durations, particularly of more than 5 years, NSCLC-specific survival improves. Potential mechanisms by which smoking cessation could improve NSCLC-specific survival include improved tolerance to initial or subsequent treatment, leading to subtle differences in disease control;^34,35^ improved treatment-response rates, which are a short-term surrogate marker of delayed disease relapse or absence of disease progression leading to increased long-term survival;^25,36–38^ and a potential pathway based on early data suggesting that nicotine might drive cancer progression through the nicotinic acetylcholine receptor.^23^ The improvement in NSCLC-specific survival indicates that abstaining from smoking now can lead to a reduced chance of dying from lung cancer if an individual is diagnosed with lung cancer at a later date. Nonetheless, from a patient perspective, overall survival, be it from lung cancer or not, will be of benefit in strengthening the public health message of smoking cessation at any timepoint.

An opportunity for emphasising the message of smoking cessation to individuals could be at lung-cancer screening, which has been found to be a cost-effective intervention to reduce lung cancer incidence and death in a simulation study in the USA.^38^ However, a systematic review and meta-analysis published in 2020^39^ reported an adherence to lung-cancer screening (ie, having participants in a screening programme return for subsequent screening) of only 55% in the USA. Furthermore, smoking relapses in participants of screening programmes occur often, even in participants motivated to quit smoking, as shown in participants enrolled in the US National Lung Screening Trial.^40^ In the future, assessing whether reinforcement messaging of the benefits of smoking abstinence and other interventions on lung-cancer survival at each screening could motivate participants to better adhere to the screening schedule and to continue to abstain from smoking might be worthwhile.

There were several limitations of this analysis. First, all smoking data were self-reported and biochemical-validation data were unavailable. This was in line with clinical practice and screening, where self-reporting is the norm. Second, the COS-ILCCO database does not have information on smoking cessation after lung-cancer diagnosis, and sparse data on treatment, targeted therapy, molecular testing, comorbidity data, socioeconomic-status (other than education), and other genetic or environmental factors which could confound the association.^41^ As an example, a person’s ability to stop smoking could be associated with adherence to treatment regimens and a healthy lifestyle, both of which might confound any relationship with overall survival. The effects of these factors on the association and size of effect is unclear. However, adjusting for comorbidities might be inappropriate anyway, as it might be both a confounder and a collider variable. Due to the observational nature, our results should be best described as clinically important associations, but causality cannot be inferred. Third, there could be some selection bias that has been introduced from the exclusion of eligible participants due to missing data. However, there were no substantial differences in clinico-demographic data between the patients who were analysed and the original dataset. Finally, there were not enough data to analyse the association with small-cell lung cancer.

We found improved overall survival in patients with NSCLC who had abstained from smoking for at least 1 year before their lung-cancer diagnosis, compared with current smokers. These associations were independent of age, sex, disease stage, and histological subtype. These findings provide evidence to increase smoking-cessation counselling, including during lung-cancer screening, and for tobacco control policies. Our results suggest that it is never too late to quit smoking.

## Supplementary Material

MMC1

## Figures and Tables

**Table 1:** Univariable and multivariable analysis of factors associated with overall survival among people who have ever smoked by Cox regression

	Univariable analysis		Multivariable analysis*	
	Crude HR (95% CI)	p value	Adjusted HR (95% CI)	p value

Age at diagnosis (>65 years *vs* ≤65 years)	1.23 (1.19–1.26)	<0.0001	1.43 (1.39–1.48)	<0.0001
Sex (female *vs* male)	0.82 (0.80–0.84)	<0.0001	0.82 (0.80–0.85)	<0.0001
Ethnicity				
Black *vs* White	0.90 (0.85–0.95)	0.0003	1.11 (1.02–1.20)	0.014
Asian *vs* White	0.63 (0.59–0.67)	<0.0001	0.88 (0.77–1.00)	0.055
Other *vs* White	0.90 (0.83–0.98)	0.012	0.84 (0.77–0.92)	0.0001
Education				
More education *vs* less education	0.78 (0.75–0.82)	<0.0001	0.83 (0.78–0.87)	<0.0001
Moderate education *vs* less education	0.91 (0.87–0.95)	<0.0001	0.91 (0.86–0.96)	0.0003
Other or missing *vs* less education	1.01 (0.97–1.06)	0.60	0.90 (0.84–0.96)	0.0011
Clinical stage at diagnosis				
2 or 3A *vs* 1	1.84 (1.78–1.91)	<0.0001	1.80 (1.73–1.87)	<0.0001
3B or 4 *vs* 1	4.49 (4.34–4.65)	<0.0001	4.69 (4.51–4.87)	<0.0001
Histology				
Squamous cell *vs* adenocarcinoma	1.11 (1.08–1.15)	<0.0001	1.13 (1.09–1.17)	<0.0001
Large cell or other *vs* adenocarcinoma	1.48 (1.43–1.53)	<0.0001	1.22 (1.17–1.26)	<0.0001
Time cohort (per increase in 10 years of diagnosis date)	1.00 (0.98–1.03)	0.86	1.06 (1.02–1.11)	0.0019
Pack years (>40 *vs* ≤40)	1.17 (1.14–1.20)	<0.0001	1.10 (1.07–1.14)	<0.0001
Smoking-abstinence duration at diagnosis				
<1 year (ie, current smokers)	1 (ref)	..	1 (ref)	..
1–3 years	0.82 (0.78–0.86)	<0.0001	0.92 (0.87–0.97)	0.0007
>3–5 years	0.89 (0.83–0.96)	0.0011	0.90 (0.83–0.97)	0.0042
>5 years	0.91 (0.88–0.93)	<0.0001	0.90 (0.87–0.93)	<0.0001

HR=hazard ratio.

* As well as the variables listed, we also adjusted for individual studies.

**Table 2:** Univariable and multivariable analysis of factors related to non-small-cell lung cancer-specific survival with a sub-distribution, hazard-function, competing risk model among people who have ever smoked

	Univariable analysis		Multivariable analysis*	
	Crude HR (95% CI)	p value	Adjusted HR (95% CI)	p value

Age at diagnosis (>65 years *vs* ≤65 years)	0.99 (0.94–1.04)	0.74	1.14 (1.08–1.22)	<0.0001
Sex (female *vs* male)	0.83 (0.79–0.88)	<0.0001	0.91 (0.85–0.98)	0.0085
Ethnicity				
Black *vs* White	0.91 (0.85–0.98)	0.015	1.02 (0.90–1.15)	0.78
Asian *vs* White	1.06 (0.98–1.14)	0.14	1.07 (0.85–1.35)	0.56
Other or missing *vs* White	1.10 (0.87–1.38)	0.44	0.84 (0.64–1.11)	0.22
Education				
More education *vs* less education	0.67 (0.62–0.72)	<0.0001	0.90 (0.80–1.00)	0.043
Moderate education *vs* less education	0.71 (0.66–0.76)	<0.0001	0.90 (0.81–0.99)	0.030
Other or missing *vs* less education	0.87 (0.80–0.95)	0.0011	1.03 (0.86–1.24)	0.73
Clinical stage at diagnosis				
2 or 3A *vs* 1	2.43 (2.25–2.63)	<0.0001	2.38 (2.20–2.58)	<0.0001
3B or 4 *vs* 1	5.51 (5.14–5.92)	<0.0001	5.55 (5.15–5.98)	<0.0001
Histology				
Squamous cell *vs* adenocarcinoma	1.12 (1.05–1.19)	0.0003	1.11 (1.04–1.19)	0.0022
Large cell or other *vs* adenocarcinoma	1.41 (1.33–1.51)	<0.0001	1.19 (1.10–1.28)	<0.0001
Time cohort (per increase in 10 years of diagnosis date)	0.96 (0.92–1.01)	0.15	0.93 (0.85–1.01)	0.077
Pack years (>40 *vs* ≤40)	1.07 (1.02–1.13)	0.0095	1.01 (0.95–1.07)	0.74
Smoking-abstinence duration at diacnosis				
<1 year (ie, current smokers)	1 (ref)	..	1 (ref)	..
1–3 years	0.80 (0.74–0.86)	<0.0001	0.94 (0.87–1.02)	0.15
>3–5 years	0.82 (0.71–0.94)	0.0041	0.91 (0.79–1.05)	0.21
>5 years	0.72 (0.68–0.77)	<0.0001	0.87 (0.81–0.93)	0.0001

HR=hazard ratio.

* As well as the variables listed, we also adjusted for individual studies.

## Data Availability

The dataset from our analysis is securely coded at the Princess Margaret Cancer Center (Toronto, ON, Canada). However, ownership of data shared within the International Lung Cancer Consortium remains with the original investigators or studies. Data-sharing agreements prohibit making the dataset publicly available, but de-identified participant data and the data dictionary will be made available upon approval by the International Lung Cancer Consortium Executive Committee and individual study principal investigators (AT, AR-R, AC, AA, AGS, CC, CH, DCC, GL, HB, HS, JKF, KS, LLM, MTL,MBS, PB, PY, RMR, SSS, YW, and JZ) via the project proposal process (https://ilcco.iarc.fr/). This analysis was approved by the same committee and followed the same mechanisms. Relevant ethical and data-sharing approval should be obtained as per International Lung Cancer Consortium policy. The statistical analysis plan and analytical code are available with publication from WX, GL, or YL upon request.

## Research in context

### Evidence before this study

We searched PubMed for all relevant studies published in English between Jan 1, 2000, and Sept 1, 2022, using the terms “smoking” OR “smoking cessation” OR “smoking abstinence” AND “lung cancer” OR “cancer”. There is a large amount of evidence of the association between smoking and lung-cancer risk, as well as many studies evaluating non-small-cell lung cancer (NSCLC) survival by smoking status at NSCLC diagnosis. However, few studies evaluated the benefits of smoking cessation before NSCLC diagnosis in terms of NSCLC survival. We identified two studies that assessed the benefit of smoking cessation before lung-cancer diagnosis, one of them indicating a statistically significant survival benefit for people who had quit smoking within 3 years of diagnosis and the other lacking a statistically significant survival benefit, possibly due to the small sample size. There were no previous large-scale studies addressing the benefits of smoking cessation on lung-cancer survival in a geographically diverse population.

### Added value of this study

To our knowledge, this is the first large-scale study to show that, in NSCLC, smoking abstinence for a duration as short as 1 year before NSCLC diagnosis was associated with improved overall survival compared with individuals who were current smokers at diagnosis. This finding was consistent across all disease stages and histologies, across both sexes, and across different cumulative smoking histories from a large-sample, pooled analysis across data from four continents (Asia, Europe, North America, and South America).

### Implications of all the available evidence

Smoking abstinence for as short as 1 year, even after a lifetime of smoking, was associated with improved NSCLC overall survival and might positively influence people who smoke to decide to quit smoking. There is evidence to increase smoking-cessation counselling, including during lung-cancer screening, and for tobacco-control policies.
