# Supplementary material for: Association between duration of smoking abstinence before non-small-cell lung cancer diagnosis and survival: a retrospective, pooled analysis of cohort studies
Source: Lancet Public Health. Author manuscript; Available in PMC 2023 Sep 29. (PMC10540150; doi:10.1016/S2468-2667(23)00131-7)

THE LANCET
Public Health

Supplementary appendix

This appendix formed part of the original submission and has been peer reviewed. We post it as supplied by the authors.

Supplement to: Fares AF, Li Y, Jiang M, et al. Association between duration of smoking abstinence before non-small-cell lung cancer diagnosis and survival: a retrospective, pooled analysis of cohort studies. *Lancet Public Health* 2023; **8:** e691–700.

Supplementary Methods

Sensitivity analyses

This study included 26 studies that are part of the International Lung Cancer Consortium (ILCCO) database. Three were cohort studies, and 23 were case-control. To address studies lacking baseline information, we performed a sensitivity analysis excluding 2,848 patients (7.5% of the pooled analysis) where smoking information was available prior to, but not at the time of, lung cancer diagnosis (n=1,819, 4.8% of the pooled analysis) or where pack-years information was lacking (n=1,029, 2.7%), as shown in **Supplementary Table 8**. For reassurance regarding the heterogeneity across studies, we performed an additional analysis, plotting all the available individual studies' hazard ratios to investigate heterogeneity for OS and NSCLC-specific survival; this is presented in **Supplementary Figure 4** and **Supplementary Figure 5**.To address individuals classified as current smokers but reporting very light smoking history (and therefore may not be truly a "current smoker"), we performed another sensitivity analysis, including only patients who self-reported smoking a minimum amount of cigarettes daily in the last year before lung cancer diagnosis or at the time of diagnosis. We decided to use the number of cigarettes smoked daily rather than pack-years, as the current-smokers definition encompasses only the last year before diagnosis, and the pack-year equation uses the cumulative number of years smoking. We evaluated the average daily number of smoked cigarettes reported to establish this cutoff, and opted to use a cut-off of 2 cigarettes per day to represent very light smoking. Next, to perform this specific sensitivity analysis for OS and NSCLC-specific survival, we excluded 41 (0.27% of total OS cohort) patients and 21 (0.22%) patients who smoked 2 cigarettes daily or less, respectively. **Supplementary Table 9** presents the OS data, while **Supplementary Table 10** presents the NSCLC-specific survival data.We performed another sensitivity analysis comparing cohort studies and case-control studies, as shown in **Supplementary Table 11.**

CONSORT Diagram

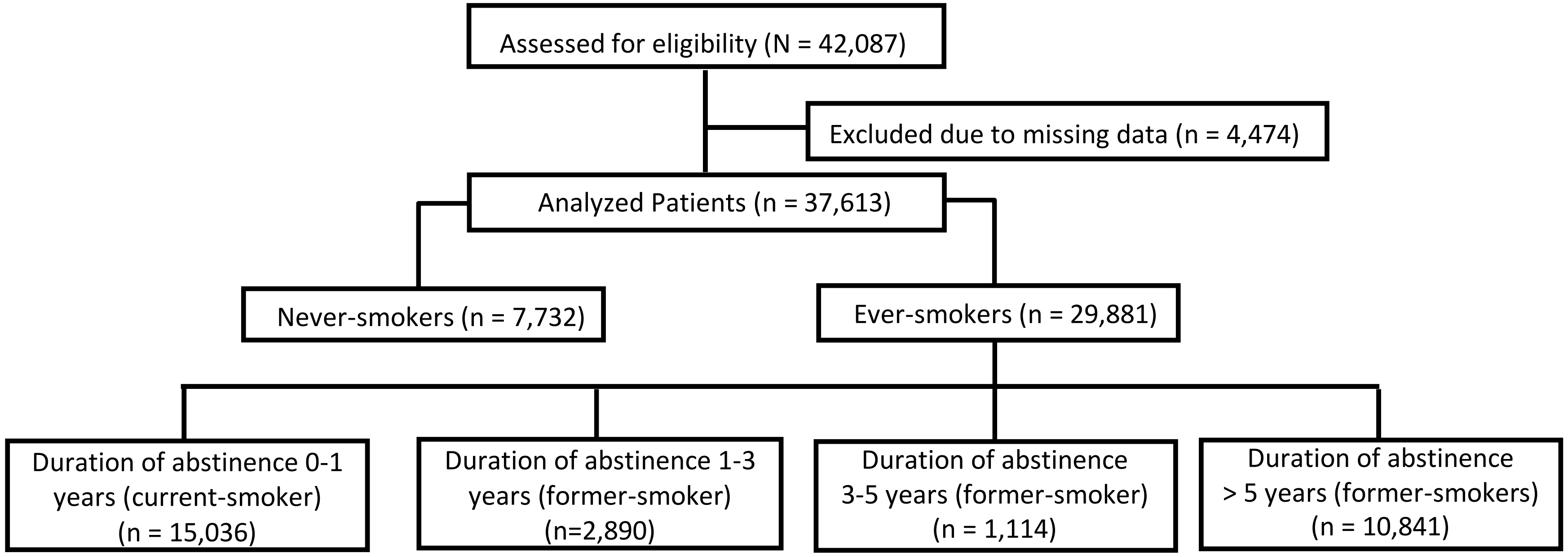

Supplementary Table 1 (Online only). Number of analyzed patients in each participating COS-ILCCO study site.

**Study name**	**Country(ies)**	**Enrollment period**	**Number of Patients**	Current Smokers	Former Smokers	Never Smokers
Mayo-EGLC	USA	1984-2016	14,458	5,509	6,675	2,274
LCS	USA	1983-2010	3,542	1,021	2,063	458
LUN MLT	Eastern Europe & Russia	2005-2016	2,262	1,253	634	375
MSH-PMH	Canada	2007-2013	2,648	782	1,167	699
FUDAN	China	2009-2014	1,721	576	185	960
NCCRI-JAPAN	Japan	2000-2016	1,494	413	330	751
EAGLE	USA	2002-2005	1,442	679	641	122
NCI-MD	USA	1998-2015	1,358	540	698	120
WHI	USA	1992-2005	1,054	629	0	425
NANJING	China	2002-2010	860	479	0	381
CARET	USA	1985-1994	765	566	197	2
TLC	USA	2001-2013	681	95	461	125
Barretos-Brazil	Brazil	2010-2020	625	387	0	238
EXHALE	USA	2005-2010	640	236	371	33
WELD	USA	2001-2005	565	206	306	53
INHALE	USA	2011-2018	465	238	184	43
DCC	USA	1991-1997	436	178	221	37
ACS-CPSII	USA	1992-2006	429	134	246	49
CAPUA	Spain	2002-2012	409	361	0	48
MDACC_LC	USA	1991-2016	348	274	0	74
LCRINS	Spain	2006-2016	328	0	0	328
LLP	England	1996-2016	318	62	196	60
FHSIII	USA	1990-2003	251	181	47	23
ReSoluCent	England	2001-2013	236	105	95	36
NELCS	USA	2005-2007	215	119	88	8
ESTHER	Germany	2000-2004	63	13	40	10
			37,613	15,036	14,845	7,732

Mayo-EGLC, Epidemiology & Genetics of Lung Cancer (Mayo Clinic); USA, United States of America; LCS, Harvard Lung Cancer Study; LUN MLT, Early stage non-small cell lung cancer study, Russian Genetic Epidemiology Study of Multiple Cancer Sites (International Agency for Research on Cancer); MSH-PMH, Multi-Cancer Case-Control Thoracic Study (Sinai Health System, Princess Margaret Cancer Centre); FUDAN, Fudan University (Shanghai); NCCRI-JAPAN, National Cancer Center Research Institute (Japan); EAGLE, Environment And Genetics in Lung cancer Etiology (National Cancer Institute, US National Institutes of Health); NCI-MD, National Cancer Institute-MD Case Control Study (US National Institutes of Health); WHI, Women’s Health Initiative (Fred Hutchinson Cancer Research Center); NANJING, Nanjing dataset (Nanjing Medical University School of Public Health); CARET, beta-Carotene And Retinol Efficacy Trial (Fred Hutchinson Cancer Research Center); TLC, Total Lung Care (Moffit Cancer Center); Barretos-Brazil (Molecular Oncology Research Center, Barretos Cancer Hospital, Barretos, Brazil); EXHALE, Exploring Health, Ancestry and Lung Epidemiology (Karmanos Cancer Institute); WELD, Women's Epidemiology of Lung Disease (Karmanos Cancer Institute); INHALE, Inflammation, Health, and Lung Epidemiology (Karmanos Cancer Institute); ACS-CPSII; American Cancer Society-Cancer Prevention Study II; DCC, Diet and Cancer Study (University of Hawaii Cancer Center); CAPUA: Cancer de Pulmón en Asturias (Universidad de Oviedo, Spain); MDACC_LC, MD Anderson-Cancer Center_Lung Cancer Study; LCRINS, Lung Cancer in Never Smokers (Universidad de Santiago de Compostela, Spain); LLP, Liverpool Lung Project (University of Liverpool); FHSIII, Family Health Study III (Karmanos Cancer Institute); ReSoluCent, Resource for the Study of Lung Cancer Epidemiology in North Trent (National Institute for Health Research, University of Sheffield); NELCS, New England Lung Cancer Study (Geisel School of Medicine at Dartmouth); ESTHER, Epidemiological study on chances of prevention, early detection and optimized therapy of chronic diseases in the older population (German Cancer-research center, Department of Clinical Epidemiology and Aging Research). (Barretos, Brazil, Barretos Cancer Hospital, Brazil).

Supplementary Table 2 (Online only). Comparison of basic clinico-demographic variables in patients included in the analysis, and those excluded because of missing primary, covariate, and outcome data. The number of variables that can be compared is smaller than in other tables, because of this missing key data. Percentages are presented in parentheses.

Variable	Categories (%), unless specified	Full dataset(n=42,087)	Analyzed patients(n=37,613)	Missing Data (n= 4,474)
Age at diagnosis	Mean (SD), years	65.4 (10.8)	65.0 (10.8)	68.7 (10.6)
Sex	Male	21,893 (52%)	20,110 (53%)	1,783 (40%)
	Female	20,194 (48%)	17,503 (47%)	2,691 (60%)
Ethnicity	White	32,191 (79%)	28,555 (78%)	3,636 (87%)
	Black	2,102 (5%)	1,936 (5%)	166 (4%)
	Asian	5,285 (13%)	4,993 (14%)	292 (7%)
	Other	1,065 (3%)	965 (3%)	100 (2%)
Education	Low	5,108 (12%)	4,661 (12%)	447 (10%)
	Moderate	11,210 (27%)	10,026 (27%)	1,184 (26%)
	High	11,392 (27%)	9,837 (26%)	1,555 (35%)
	Other/missing	14,377 (34%)	13,089 (35%)	1,288 (29%)
Histology	Adenocarcinoma	24,894 (59%)	22,216 (59%)	2,678 (60%)
	Squamous carcinoma	10,088 (24%)	9,084 (24%)	1,004 (22%)
	Large cell carcinoma/Other	7,105 (17%)	6,313 (17%)	792 (18%)
Clinical Stage at Diagnosis	I	12,873 (31%)	11,559 (31%)	1,314 (30%)
	II/IIIA	11,107 (26%)	10,055 (27%)	1,125 (26%)
	IIIB/IV	17,956 (43%)	15,999 (43%)	1,958 (45%)
Pack-Years	Less than forty packs per year	13,251 (44%)	11,885 (43%)	1,366 (52%)
	More than forty packs per year	16,762 (56%)	15,506 (57%)	1,256 (48%)
Time-cohort	Mean (SD), in ten-years of diagnosis date	200.6 (0.6)	200.6 (0.6)	200.8 (0.6)

Supplementary Table 3. Demographics, clinical characteristics, and smoking information by smoking status and categories of duration of abstinence (among former smokers).

Variable	Categories N (%), unless specified	Current Smokers (n=15,036)	Former smokers (n=14,845)	Former smokers: Period of abstinence			Never Smokers (n=7732)
				Quit between 1-3 years (n=2,890)	Quit between 3-5 years (n=1,114)	Quit over 5 years (n=10,841)	
Age at diagnosis	Median (IQR), years	63 (14)	69 (13)	64 (13)	66 (13)	71 (12)	64 (18)
Sex	Males	8,929 (59)	9,011 (61)	1,661 (57)	649 (58)	6,701 (62)	2,170 (28)
	Females	6,107 (41)	5,834 (39)	1,229 (43)	465 (42)	4,140 (38)	5,562 (72)
Ethnicity	White	11,422 (79)	12,562 (87)	2,228 (78)	914 (85)	9,420 (89)	4,571 (60)
	Black	898 (6)	823 (6)	305 (11)	65 (6)	453 (4)	215 (3)
	Asian	1,619 (11)	756 (5)	251 (9)	73 (7)	432 (4)	2,618 (34)
	Other	459 (3)	319 (2)	68 (2)	28 (3)	223 (2)	187 (2)
Education	Low	2,068 (14)	1,673 (11)	423 (15)	139 (12)	1,111 (10)	920 (12)
	Moderate	4,534 (30)	4,224 (28)	969 (34)	340 (31)	2,915 (27)	1,268 (16)
	High	3,560 (24)	4,190 (28)	807 (28)	317 (28)	3,066 (28)	2,087 (27)
	Other/missing	4,874 (32)	4,758 (32)	691 (24)	318 (29)	3,749 (35)	3,457 (45)
Histology	Adenocarcinoma	7,360 (49)	8,404 (57)	1,435 (50)	555 (50)	6,414 (59)	6,452 (83)
	Squamous cell	4,630 (31)	3,915 (26)	893 (31)	352 (32)	2,670 (25)	539 (7)
	Large cell/Other	3,046 (20)	2,526 (17)	562 (19)	207 (19)	1,757 (16)	741 (10)
Clinical stage at diagnosis	I	4,071 (27)	4,777 (32)	920 (32)	353 (32)	3,504 (32)	2,551 (33)
	II/IIIA	4,375 (29)	4,037 (27)	862 (30)	308 (28)	2,867 (26)	1,570 (21)
	IIIB/IV	6,439 (43)	6,030 (41)	1,107 (38)	453 (41)	4,470 (41)	3,529 (46)
Smoking duration	Median (IQR), years	42 (15)	35 (18)	43 (14)	41 (16)	30 (20)	Not applicable
Cigarettes per day	Median (IQR), n	20 (10)	20 (10)	20 (11)	20 (15)	20 (13)	Not applicable
Pack-Years	Median (IQR)	46 (32)	38 (36)	47 (34)	49 (37)	34 (32)	Not applicable

Supplementary Table 4 (Online only). Comparison of numbers of patients providing education and ethnicity variables, among ever-smokers included in multivariable analyses. Note the large number of Asians with missing education variables, which is due to the fact that the studies from the Asian continent did not collect such data. Row percentages are presented in parentheses.

**Ethnicity**	**Education**				**Total**
	**Low**	**Moderate**	**High**	**Missing**	
White	3,014 (14)	6,554 (30)	6,087 (28)	6,323 (29)	21,978 (100)
Black	160 (10)	795 (48)	577 (35)	120 (7)	1,652 (100)
Asian	154 (7)	285 (12)	188 (8)	1,703 (73)	2,330 (100)
Other/missing	112 (16)	219 (31)	167 (23)	219 (31)	717 (100)
**Total**	3,440 (13)	7,852 (29)	7,019 (26)	8,365 (31)	26,677(100)

Supplementary Table 5 (Online only): Univariable and Multivariable analysis of factors associated with overall survival, by Cox regression, among individuals of all smoking status (never-, former-, current-smokers).

**Variables**	**Categories**	**Univariable analysis**		**Multivariable analysis***	
		**Unadjusted HR (95%CI)**	**P value**	**Adjusted HR (95%CI)**	**P value**
**Age At diagnosis**	**Over 65 years *versus* 65 years and under**	1.26 (1.23,1.29)	**<0.0001**	1.45 (1.41,1.48)	**<0.0001**
**Sex**	**Females *versus* Males**	0.76 (0.75,0.78)	**<0.0001**	0.82 (0.80,0.84)	**<0.0001**
**Ethnicity**	**Black *versus* White**	0.92 (0.87,0.97)	**0.0020**	1.05 (0.97,1.12)	0.2242
	**Asian *versus* White**	0.49 (0.47,0.52)	**<0.001**	0.85 (0.77,0.92)	**0.0002**
	**Other/missing *versus* White**	0.89 (0.83,0.96)	**0.0035**	0.84 (0.78,0.91)	**<0.0001**
**Education**	**High *versus* Low**	0.75 (0.72,0.79)	**<0.0001**	0.82 (0.78,0.86)	**<0.0001**
	**Moderate *versus* Low**	0.88 (0.85,0.92)	**<0.0001**	0.91 (0.86,0.95)	**<0.0001**
	**Other/missing *versus* Low**	0.86 (0.83,0.9)	**<0.0001**	0.91 (0.86,0.96)	**0.0004**
**Clinical stage at diagnosis**	**II/IIIA *versus* I**	2.03 (1.96,2.10)	**<0.0001**	1.93 (1.86,2.00)	**<0.0001**
	**IIIB/IV *versus* I**	4.86 (4.71,5.02)	**<0.0001**	5.02 (4.85,5.20)	**<0.0001**
**Histology**	**Squamous cell *versus* Adenocarcinoma**	1.22 (1.18,1.25)	**<0.0001**	1.17 (1.13,1.21)	**<0.0001**
	**Large cell/Other *versus* Adenocarcinoma**	1.60 (1.55,1.65)	**<0.0001**	1.24 (1.20,1.29)	**<0.0001**
**Time-cohort**	**Per increase in ten-years of diagnosis date**	0.94 (0.92,0.97)	**<0.0001**	0.99 (0.95,1.02)	0.3793
**Smoking status at diagnosis**	**Former smokers *versus* current smokers**	0.89 (0.86,0.91)	**<0.0001**	0.88 (0.86,0.91)	**<0.0001**
	**Never smokers *versus* current smokers**	0.66 (0.64,0.69)	**<0.0001**	0.73 (0.70,0.76)	**<0.0001**

Supplementary Table 6 (Online only). Overall and non-small cell lung cancer (NSCLC) survival estimates at 5 years and 10 years by smoking status (top table) and in former smokers by smoking cessation duration (bottom table) for a prototypical patient. For these estimates, we had to make specific assumptions about the prototypical individual for which these estimates apply. We utilized the most frequent or median category for each of our assumptions. Our assumptions were: age category (<65 years), gender (male), ethnicity (white), educational level (low), stage at diagnosis (I), histology (adenocarcinoma), diagnosis year (2005), pack-years (<40 pack-years) and study site (Mayo Clinic).

		Percentage alive (95% confidence interval)		
**Survival outcome**		**Current smoker**	**Former smoker**	**Never smoker**
**5 Year**	**Proportion alive (overall survival)**	53% (51-55%)	57% (55-59%)	63% (61-65%)
**10 Year**		35% (33-37%)	40% (37-42%)	46% (44-49%)
**5 Year**	**Cumulative incidence of death from NSCLC**	21% (13-28%)	19% (12-26%)	17% (11-23%)
**10 Year**		26% (17-35%)	23% (15-31%)	21% (13-28%)

**Survival outcome**		**Current smoker**	**Quit 1-3 years ago**	**Quit 3-5 years ago**	**Quit >5 years ago**
**5 Year**	**Proportion alive (overall survival)**	54% (51-56%)	57% (54-59%)	57% (54-61%)	57% (55-60%)
**10 Year**		36% (33-39%)	39% (36-42%)	40% (36-44%)	40% (37-43%)
**5 Year**	**Cumulative incidence of death from NSCLC**	27% (24-30%)	26% (22-29%)	25% (21-29%)	24% (21-27%)
**10 Year**		34% (30-37%)	32% (28-36%)	31% (26-36%)	30% (26-33%)

Supplementary Table 7 (Online only). Baseline clinical prognostic multivariable Cox-proportional hazard models of clinico-demographic factors associated with overall survival in non-small cell lung cancer patients, among ever-smokers. The variable, duration of abstinence, is specifically not included in these models.

Variables	Categories	Analysis excluding Smoking Status		Analysis including Smoking Status	
		Adjusted HR (95% CI)	P value	Adjusted HR (95%CI)	P value
**Age At diagnosis**	**Over 65 years *versus* 65 years and under**	1.39 (1.35,1.43)	**<0.0001**	1.43 (1.38,1.47)	<**0.0001**
**Sex**	**Females *versus* Males**	0.83 (0.81,0.86)	**<0.0001**	0.82 (0.80,0.85)	<**0.0001**
**Ethnicity**	**Black *versus* White**	1.12 (1.04,1.22)	**0.0050**	1.11 (1.02,1.20)	**0.01314**
	**Asian *versus* White**	0.89 (0.78,1.02)	0.0844	0.88 (0.77,1.00)	0.05759
	**Other/missing *versus* White**	0.85 (0.78,0.93)	**0.0002**	0.84 (0.77,0.92)	**0.0001**
**Education**	**High *versus* Low**	0.82 (0.78,0.87)	**<0.0001**	0.83 (0.78,0.87)	<**0.0001**
	**Moderate *versus* Low**	0.9 (0.86,0.95)	**0.0002**	0.91 (0.86,0.96)	**0.0003**
	**Other/missing *versus* Low**	0.89 (0.84,0.95)	**0.0005**	0.90 (0.84,0.96)	**0.0010**
**Clinical stage at diagnosis**	**II/IIIA *versus* I**	1.80 (1.73,1.87)	**<0.0001**	1.80 (1.73,1.87)	<**0.0001**
	**IIIB/IV *versus* I**	4.70 (4.53,4.88)	**<0.0001**	4.69 (4.51,4.87)	<**0.0001**
**Histology**	**Squamous cell *versus* Adenocarcinoma**	1.14 (1.10,1.17)	**<0.0001**	1.13 (1.09,1.17)	<**0.0001**
	**Large cell/Other *versus* Adenocarcinoma**	1.22 (1.18,1.27)	**<0.0001**	1.22 (1.17,1.26)	<**0.0001**
**Time-cohort**	**Per increase in ten-years of diagnosis date**	1.07 (1.03,1.11)	**0.0010**	1.06 (1.02,1.11)	**0.0020**
**Pack-years**	**More than *versus* at most forty**	1.13 (1.10,1.16)	**<0.0001**	1.10 (1.07,1.14)	**<0.0001**
**Smoking Status**	**Former- *versus* Current-smoker**	not applicable		0.90 (0.87,0.93)	<**0.0001**

* Multivariable analysis was adjusted for age, sex, ethnicity, education, stage, histology, diagnosis year and packyear, where applicable; HR, Hazard Ratio; CI, confidence interval

Supplementary Table 8 (Online only). Sensitivity analysis, excluding the studies lacking smoking status information at lung cancer diagnosis or missing pack-years, showing the demographics, clinical characteristics, and self-reported smoking information by smoking status and categories of duration of abstinence (among former smokers).

Variables	Categories	Univariable analysis		Multivariable analysis	
		Adjusted HR (95% CI)	P value	Adjusted HR (95% CI)	P value
**Age at diagnosis**	**Over 65 years *versus* 65 years and under**	1.21 (1.18,1.24)	**<0.0001**	1.43 (1.39,1.48)	**<0.0001**
**Sex**	**Females *versus* Males**	0.79 (0.77,0.82)	**<0.0001**	0.82 (0.80,0.85)	**<0.0001**
**Ethnicity**	**Black *versus* White**	0.88 (0.83,0.94)	**<0.001**	1.10 (1.01,1.20)	0.0246
	**Asian *versus* White**	0.85 (0.80,0.90)	**<0.001**	0.88 (0.77,1.01)	0.0595
	**Other/missing *versus* White**	0.89 (0.82,0.96)	**0.0047**	0.84 (0.77,0.91)	**<0.0001**
**Education**	**High *versus* Low**	0.78 (0.75,0.82)	**<0.0001**	0.83 (0.78,0.88)	**<0.001**
	**Moderate *versus* Low**	0.92 (0.88,0.96)	**0.0004**	0.91 (0.86,0.96)	**0.0006**
	**Other/missing *versus* Low**	1.03 (0.99,1.08)	0.1329	0.89 (0.83,0.95)	**0.0005**
**Clinical stage at diagnosis**	***II/IIIA versus I***	1.87 (1.80,1.94)	**<0.0001**	1.80 (1.72,1.87)	**<0.0001**
	**IIIB/IV *versus* I**	4.44 (4.28,4.60)	**<0.0001**	4.71 (4.54,4.90)	**<0.0001**
**Histology**	**Squamous cell versus Adenocarcinoma**	1.12 (1.09,1.16)	**<0.0001**	1.13 (1.09,1.17)	**<0.0001**
	**Large cell/Other versus Adenocarcinoma**	1.48 (1.43,1.53)	**<0.0001**	1.21 (1.17,1.26)	**<0.0001**
**Time-cohort**	**Per increase in ten-years of diagnosis date**	1.03 (1.00,1.06)	0.0224	1.07 (1.03,1.12)	**0.0014**
**Pack-Years**	**More than *versus* at most forty**	1.16 (1.12,1.19)	**<0.0001**	1.10 (1.06,1.13)	**<0.001**
**Smoking status at diagnosis**	**Current smokers**				
	**Period of abstinence 1-3 years**	0.79 (0.76,0.83)	**<0.0001**	0.91 (0.87,0.96)	**0.0005**
	**Period of abstinence 3-5 years**	0.85 (0.79,0.91)	**<0.0001**	0.88 (0.82,0.95)	**0.0012**
	**Period of abstinence >5 years**	0.88 (0.86,0.91)	**<0.0001**	0.89 (0.86,0.93)	**<0.0001**

Supplementary Table 9 (Online only). Sensitivity analysis using overall survival as outcome, excluding current smokers who self-reported as smoking less than 2 cigarettes daily at lung cancer diagnosis. We removed 41 patients in total.

Variables	Categories	Univariable analysis		Multivariable analysis	
		Adjusted HR (95% CI)	P value	Adjusted HR (95% CI)	P value
**Age at diagnosis**	**Over 65 years *versus* 65 years and under**	1.23 (1.19,1.26)	**<0.0001**	1.43 (1.39,1.48)	**<0.0001**
**Sex**	**Females *versus* Males**	0.82 (0.80,0.84)	**<0.0001**	0.82 (0.80,0.85)	**<0.0001**
**Ethnicity**	**Black *versus* White**	0.90 (0.85,0.95)	**0.0003**	1.11 (1.02,1.20)	**0.0135**
	**Asian *versus* White**	0.63 (0.59,0.67)	**<0.0001**	0.88 (0.77,1.01)	0.0611
	**Other/missing *versus* White**	0.90 (0.83,0.98)	**0.0119**	0.84 (0.77,0.92)	**0.0001**
**Education**	**High *versus* Low**	0.78 (0.75,0.82)	**<0.0001**	0.83 (0.78,0.87)	**<0.0001**
	**Moderate *versus* Low**	0.91 (0.87,0.95)	**<0.0001**	0.91 (0.86,0.96)	**0.0002**
	**Other/missing *versus* Low**	1.01 (0.99,1.06)	0.6026	0.90 (0.84,0.96)	**0.0011**
**Clinical stage at diagnosis**	***II/IIIA versus I***	1.85 (1.78,1.92)	**<0.0001**	1.80 (1.73,1.87)	**<0.0001**
	**IIIB/IV *versus* I**	4.50 (4.35,4.66)	**<0.0001**	4.69 (4.52,4.87)	**<0.0001**
**Histology**	**Squamous cell versus Adenocarcinoma**	1.11 (1.08,1.15)	**<0.0001**	1.13 (1.09,1.17)	**<0.0001**
	**Large cell/Other versus Adenocarcinoma**	1.48 (1.43,1.53)	**<0.0001**	1.22 (1.17,1.26)	**<0.0001**
**Time-cohort**	**Per increase in ten-years of diagnosis date**	1.00 (0.98,1.03)	0.8780	1.06 (1.02,1.11)	**0.0023**
**Pack-Years**	**More than *versus* at most forty**	1.17 (1.14,1.21)	**<0.0001**	1.10 (1.07,1.13)	**<0.0001**
**Smoking status at diagnosis**	**Current smokers**				
	**Period of abstinence 1-3 years**	0.82 (0.78,0.86)	**<0.0001**	0.92 (0.87,0.97)	**0.0006**
	**Period of abstinence 3-5 years**	0.89 (0.83,0.95)	**0.0010**	0.90 (0.83,0.97)	**0.0039**
	**Period of abstinence >5 years**	0.91 (0.88,0.93)	**<0.0001**	0.90 (0.87,0.93)	**<0.0001**

Supplementary Table 10 (Online only). Sensitivity analysis using lung-cancer-specific-survival as outcome, excluding current smokers who self-reported as smoking less than 2 cigarettes daily at lung cancer diagnosis. We removed 21 patients in total.

Variables	Categories	Univariable analysis		Multivariable analysis*	
		Crude HR (95%CI)	P value	Adjusted HR (95%CI)	P value
**Age at diagnosis**	**Over 65 years *versus* 65 years and under**	0.99 (0.94,1.04)	0.7680	1.15 (1.08,1.22)	**<0.0001**
**Sex**	**Females *versus* Males**	0.83 (0.79,0.88)	**<0.0001**	0.91 (0.85,0.98)	**0.0090**
**Ethnicity**	**Black *versus* White**	0.91 (0.85,0.98)	**0.0165**	1.02 (0.90,1.15)	0.7857
	**Asian *versus* White**	1.06 (0.98,1.14)	0.1394	1.08 (0.86,1.37)	0.4966
	**Other/missing *versus* White**	1.09 (0.87,1.38)	0.4445	0.84 (0.64,1.11)	0.2169
**Education**	**High *versus* Low**	0.67 (0.62,0.72)	**<0.0001**	0.89 (0.80,1.00)	**0.0418**
	**Moderate *versus* Low**	0.71 (0.66,0.76)	**<0.0001**	0.89 (0.81,0.99)	**0.0259**
	**Other/missing *versus* Low**	0.87 (0.80,0.95)	**0.0011**	1.03 (0.86,1.23)	0.7467
**Clinical stage at diagnosis**	***II/IIIA versus I***	2.44 (2.26,2.64)	**<0.0001**	2.40 (2.21,2.60)	**<0.0001**
	**IIIB/IV *versus* I**	5.53 (5.15,5.94)	**<0.0001**	5.57 (5.17,6.01)	**<0.0001**
**Histology**	**Squamous cell *versus* Adenocarcinoma**	1.12 (1.05,1.19)	**0.0003**	1.11 (1.04,1.19)	**0.0025**
	**Large cell/Other *versus* Adenocarcinoma**	1.41 (1.32,1.51)	**<0.0001**	1.19 (1.10,1.28)	**<0.001**
**Time-cohort**	**Per increase in ten-years of diagnosis date**	0.96 (0.92,1.01)	0.1430	0.93 (0.85,1.01)	0.0707
**Pack-years**	**More than *versus* at most forty**	1.07 (1.02,1.13)	**0.0115**	1.01 (0.95,1.07)	0.8015
**Smoking abstinence duration at diagnosis**	**Current smokers (0-<1 year)**	Reference		Reference	
	**Period of abstinence 1-3 years**	0.80 (0.74,0.86)	**<0.0001**	0.94 (0.86,1.02)	0.1340
	**Period of abstinence >3-5 years**	0.82 (0.71,0.94)	**0.0038**	0.91 (0.79,1.05)	0.2028
	**Period of abstinence >5 years**	0.72 (0.68,0.77)	**<0.0001**	0.86 (0.80,0.93)	**<0.0001**

Supplementary Table 11 (Online only). Sensitivity analysis comparing results by study design. Out of 26 studies, 23 were case-control and 3 were cohort studies. The results are consistent with our primary results.

**Outcome**	**Study type**	**N (N events)**	**HR (95% CI)**
**Overall survival**	**Case-control**	24,901 (18,657)	0.90 (0.87-0.93)
	**Cohort**	1,776 (1,521)	0.93 (0.80-1.07)
	**All**	26,677 (20,178)	0.90 (0.87- 0.93)
**NSCLC-specific survival**	**Case-control**	8120 (4770)	0.86 (0.81-0.92)
	**Cohort**	1133 (734)	1.08 (0.93-1.27)
	**All**	9,253 (5,504)	0.90 (0.84-0.95)

Supplementary Figure 1 (Online only): Forest plots of subgroup multivariable analyses by different cumulative smoking (pack-years) categories, comparing overall survival hazard ratios of former-smokers (with categorized durations of abstinence prior to NSCLC diagnosis) *versus* (*vs.*) current-smokers.

Adjusted Hazard Ratios (95% Confidence Intervals) presented in tabular form and forest plots

Sub-population	N (evens)	Former smokers quit 1-3 years ago	vs.	Current smokers	Former smokers quit 3-5 years ago	vs.	Current smokers	Former smokers quit >5 years ago	vs.	Current smokers
Pack-years <20	4006 (2799)	0.86 (0.73, 1.02)	0.0760		0.63 (0.49, 0.81)	0.0003		0.83 (0.75, 0.92)	0.0004	
Pack-years >=20	26670 (17379)	0.93 (0.88, 0.98)	0.0046		0.93 (0.86, 1.00)	0.0503		0.90 (0.86, 0.93)	<0.0001	
Pack-years <30	7336 (5238)	0.87 (0.78, 0.98)	0.0189		0.80 (0.68, 0.94)	0.0080		0.88 (0.82, 0.94)	0.0003	
Pack-years >=30	19340 (14940)	0.94 (0.89, 0.99)	0.0196		0.92 (0.85, 1.00)	0.0533		0.90 (0.86, 0.94)	<0.0001	
Pack-years <40	11597 (8305)	0.89 (0.82, 0.97)	0.0085		0.81 (0.71, 0.92)	0.0012		0.88 (0.83, 0.93)	<0.0001	
Pack-years >=40	15079 (11873)	0.94 (0.88, 0.99)	0.0321		0.95 (0.86, 1.04)	0.2290		0.91 (0.87, 0.95)	<0.0001	
				0.71 – 1.1<br>HR (95% CI)			0.50 – 1.2<br>HR (95% CI)			0.71 – 1.2<br>HR (95% CI)

Supplementary Figure 2 (Onine only): Forest plots of multivariable competing risk models using subdistribution adjusted hazard functions, by different cumulative smoking (pack-years) categories, comparing NSCLC specific survival of former smokers (with categorized durations of abstinence prior to NSCLC diagnosis) *versus* (*vs.*) current-smokers.

Adjusted Hazard Ratios (95% Confidence Intervals) pr esented in tabular form and forest plots

Sub-population	N (evens)	Former smokers quit 1-3 years ago	vs.	Current smokers	Former smokers quit 3-5 years ago	vs.	Current smokers	Former smokers quit >5 years ago	vs.	Current smokers
Pack-years <20	1573 (867)	0.88 (0.68, 1.13)	0.3205		0.63 (0.40, 1.00)	0.0502		0.79 (0.65, 0.95)	0.0129	
Pack-years >=20	7679 (4637)	0.96 (0.88, 1.05)	0.3607		0.97 (0.83, 1.13)	0.6769		0.90 (0.83, 0.97)	0.0079	
Pack-years <30	2855 (1622)	0.90 (0.76, 1.06)	0.2068		0.82 (0.60, 1.11)	0.1901		0.79 (0.70, 0.90)	0.0004	
Pack-years >=30	6397 (3882)	0.96 (0.87, 1.05)	0.3504		0.96 (0.82, 1.13)	0.6571		0.92 (0.84, 1.00)	0.0635	
Pack-years <40	4419 (2549)	0.94 (0.83, 1.07)	0.3536		0.87 (0.70, 1.09)	0.2294		0.84 (0.76, 0.93)	0.0008	
Pack-years >=40	4833 (2955)	0.94 (0.84, 1.04)	0.2348		0.94 (0.79, 1.13)	0.5344		0.91 (0.81, 1.01)	0.0803	

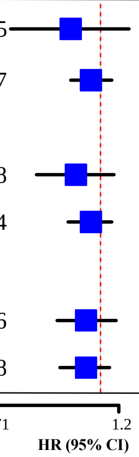

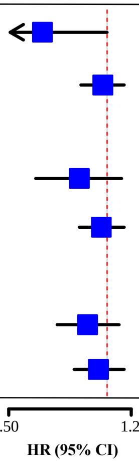

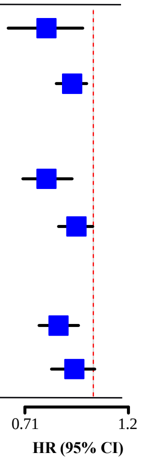

Supplementary Figure 3 (Online only). Forest plots comparing smoking cessation survival benefits by the decade of lung cancer diagnosis. There is a larger benefit observed for the recent decades, both in OS and NSCLS-specific survival. For NSCLC-specific-survival, 1980s and 1990s were merged due to small sample size of both.

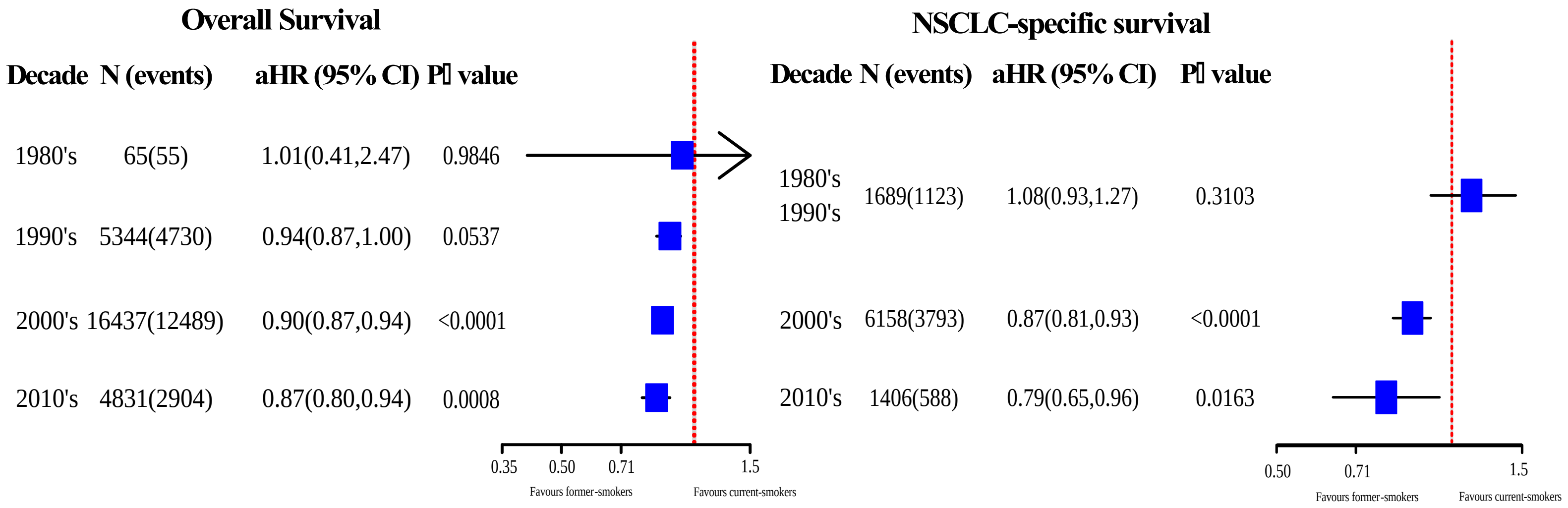

Supplementary Figure 4 (Online only). Forest plot showing the adjusted hazard ratio (aHR) for studies included in the Overall Survival analysis. Here we found a heterogeneity-test I2=19.17%, Q=22.27, p=0.22, indicating low heterogeneity. We found a meta-analytic aHR of 0.90 (CI 95% 0.86-0.94) using random effects model, similar to our original Cox model aHR of 0.90 (0.87-0.93) reported in Figure 2. Only 19 studies are shown. We removed the following seven studies: two studies did not provide pack-years information; one study included only never-smokers; four studies did not include duration of smoking abstinence information.

Forest plot of adjusted HR among study sites (OS)

Study sites	N (events)	HR (95% CI)	P–value
ILCCO–06–NIH	1193(832)	0.93(0.8,1.07)	0.3059
ILCCO–09–HARVARD	2959(2168)	0.97(0.88,1.07)	0.5917
ILCCO–15–WELD	512(392)	0.77(0.62,0.94)	0.0126
ILCCO–16–FHSIII	222(191)	1.06(0.72,1.55)	0.7771
ILCCO–17–Hawaii	385(368)	0.92(0.74,1.15)	0.4706
ILCCO–20–LIVERPOOL	250(182)	1.33(0.92,1.93)	0.1314
ILCCO–22–CARET	761(661)	1.02(0.85,1.22)	0.8305
ILCCO–23–MAYO	10475(9057)	0.91(0.87,0.95)	<0.0001
ILCCO–24–NELCS	205(144)	0.91(0.63,1.32)	0.6251
ILCCO–27–Woll	195(187)	0.84(0.59,1.17)	0.3014
ILCCO–30–Brenner	53(47)	0.61(0.29,1.27)	0.1878
ILCCO–38–Eagle	1288(1008)	0.89(0.78,1.01)	0.0729
ILCCO–48–Toronto	1736(1201)	0.79(0.7,0.9)	0.0002
ILCCO–54–IARC	1877(952)	0.92(0.79,1.06)	0.2319
ILCCO–57–ACS_CPSII	372(318)	1.08(0.84,1.4)	0.5265
ILCCO–59–EXHALE	606(473)	0.84(0.69,1.02)	0.0739
ILCCO–72–INHALE	418(171)	0.73(0.51,1.03)	0.0731
ILCCO–73–Japan	742(438)	0.84(0.69,1.03)	0.0915
ILCCO–75–FUDAN	753(80)	0.69(0.39,1.24)	0.2154
Overall		0.90(0.86,0.94)	

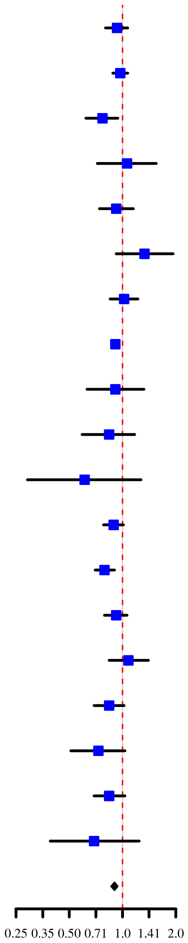

Supplementary Figure 5 (Online only). Forest plot showing the adjusted hazard ratios (aHR) for studies included in the NSCLC-specific survival analysis. Here we found a heterogeneity-test I^2= 68.13%, Q= 34.52, P-value for Q= 0.0003, defining moderate to substantial heterogeneity. It is important to notice that this heterogeneity is still acceptable once we have adjusted for the study sites in the multivariable analyses. We found a meta-analytic aHR of 0.91 (CI 95% 0.81-1.03) using the random effects model, similar to our original Cox model aHR of 0.90 (0.84-0.95) reported in Figure 2.

Forest plot of adjusted HR among study sites (CSS)

Study sites	N (events)	HR (95% CI)	P–value
ILCCO–06–NIH	1031(590)	0.93(0.78,1.12)	0.4564
ILCCO–15–WELD	512(287)	0.77(0.6,0.98)	0.0376
ILCCO–16–FHSIII	222(166)	1.41(0.97,2.06)	0.0712
ILCCO–20–LIVERPOOL	249(152)	1.75(1.14,2.71)	0.0111
ILCCO–22–CARET	761(530)	1.05(0.89,1.25)	0.5579
ILCCO–30–Brenner	53(32)	0.36(0.17,0.74)	0.0058
ILCCO–38–Eagle	1281(911)	0.89(0.8,0.99)	0.0351
ILCCO–48–Toronto	1695(890)	0.75(0.65,0.87)	0.0001
ILCCO–57–ACS_CPSII	372(204)	1.09(0.81,1.46)	0.5914
ILCCO–59–EXHALE	606(358)	0.79(0.63,0.99)	0.0450
ILCCO–72–INHALE	418(117)	0.83(0.58,1.19)	0.0306
ILCCO–73–Japan	742(418)	0.84(0.68,1.04)	0.1031
Overall		0.91(0.81,1.03)	

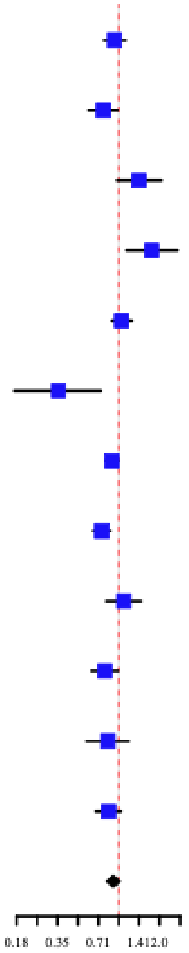

Supplementary Figure 6 (Online only). Simplified Directed Acyclic Graph describing potential confounding and collider variables for the relationship between smoking cessation (described here in its inverse form, i.e. continued smoking) and lung cancer mortality. This study describes important public health relevant associations but cannot ascribe definitive causality to these relationships because some variables were not measured (superscript "a") while others were only partly measured (superscript "b"). Most of these variables are expected to have very modest effect on the main relationships, and should not detract from the main public health message**.** Comorbidities, such as cardiovascular and chronic respiratory disease, however, could be both a confounder and a collider.

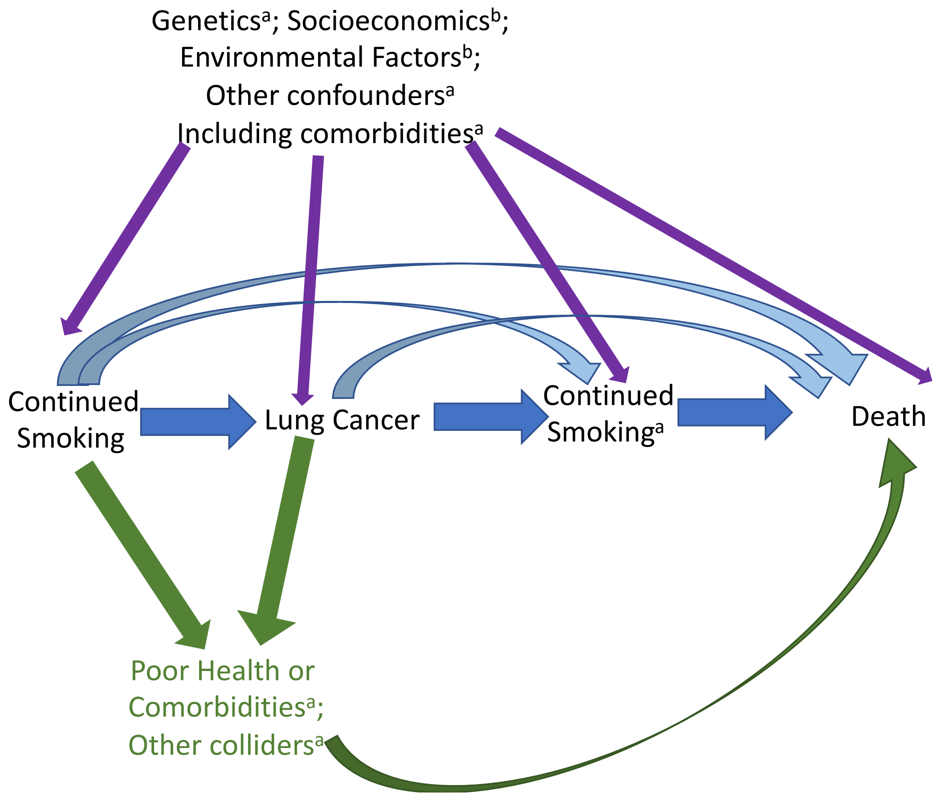

Supplement: MMC1 [file NIHMS1929205-supplement-MMC1.pdf]
